# Dipeptidyl peptidase-4 inhibitors and fracture risk: an updated meta-analysis of randomized clinical trials

**DOI:** 10.1038/srep29104

**Published:** 2016-07-07

**Authors:** Jianying Fu, Jianhong Zhu, Yehua Hao, Chongchong Guo, Zhikun Zhou

**Affiliations:** 1 Department of Pharmacy, Guangdong Medical University, No. 1, Xincheng Dadao, Songshan Lake Science and Technology Industry Park, Dongguan 523808, China; 2 Department of Pharmacy, Sun Yat-Sen Memorial Hospital, Guangzhou 510120, China

## Abstract

Data on the effects of dipeptidyl peptidase-4 (DPP-4) inhibitors on fracture risk are conflicting. Here, we performed a systematic review and meta-analysis of randomized controlled trials (RCTs) assessing the effects of DPP-4 inhibitors. Electronic databases were searched for relevant published articles, and unpublished studies presented at ClinicalTrials.gov were searched for relevant clinical data. Eligible studies included prospective randomized trials evaluating DPP-4 inhibitors versus placebo or other anti-diabetic medications in patients with type 2 diabetes. Study quality was determined using Jadad scores. Statistical analyses were performed to calculate the risk ratios (RRs) and 95% confidence intervals (CIs) using fixed-effects models. There were 62 eligible RCTs with 62,206 participants, including 33,452 patients treated with DPP-4 inhibitors. The number of fractures was 364 in the exposed group and 358 in the control group. The overall risk of fracture did not differ between patients exposed to DPP-4 inhibitors and controls (RR, 0.95; 95% CI, 0.83–1.10; P = 0.50). The results were consistent across subgroups defined by type of DPP-4 inhibitor, type of control, and length of follow-up. The study showed that DPP-4 inhibitor use does not modify the risk of bone fracture compared with placebo or other anti-diabetic medications in patients with type 2 diabetes.

Type 2 diabetes is a highly prevalent disease, especially in elderly and obese patients. Cumulative evidence shows that type 2 diabetes is associated with an increased risk of bone fracture^1^,^2^. Several anti-diabetes drugs have been reported to increase the incidence of fractures^3^,^4^.

Dipeptidyl peptidase-4 (DPP-4) inhibitors, a class of incretin based agents for the treatment of type 2 diabetes, have intermediate efficacy regarding glucose control with a satisfactory tolerability profile^5^,^6^,^7^. Data on the effects of DPP-4 inhibitors on fracture risk are conflicting. A meta-analysis of randomized controlled trials (RCTs) suggested that DPP-4 inhibitors reduced the risk of bone fracture^8^. However, a recent retrospective population-based cohort study concluded that DPP-4 inhibitors were not associated with fracture risk compared with controls and other non-insulin anti-diabetic drugs (NIADs)^9^.

The association between DPP-4 inhibitors and the risk of fracture in patients with type 2 diabetes has not been well established. We therefore performed a meta-analysis of randomized trials to provide a more robust answer regarding the risk of fracture in patients with type 2 diabetes treated with DPP-4 inhibitors.

## Results

### Search results

**Figure 1:**
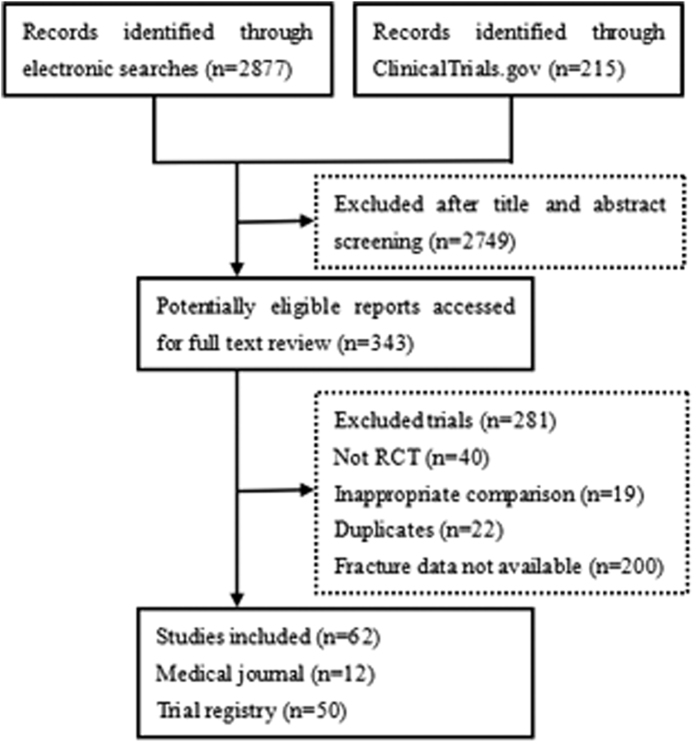
Trial flow diagram.

A total of 3092 unique titles and abstracts were identified in initial searches of the electronic database. After screening titles and abstracts, we retrieved 343 reports for full text screening. A total of 62 RCTs, including 13 from journals^10^,^11^,^12^,^13^,^14^,^15^,^16^,^17^,^18^,^19^,^20^,^21^,^22^,^23^ and 49 from the trial registry (available from https://clinicaltrials.gov) were included in the final analysis. The details of the study selection flow are described in Fig. 1.

### Study characteristics

The baseline characteristics of trials are included in Table 1 and the quality assessment results are listed in Table S1. A total of 62,206 patients (33,452 in the experimental group and 28,754 in the control group) were included in this analysis, of which 722 had fractures (364 in the experimental group and 358 in the control group). The age of the included patients ranged from 49.7 to 74.9 years. The inhibitors tested in the trials were alogliptin in 7, linagliptin in 13, saxagliptin in 9, sitagliptin in 27, anagliptin in 1, and vildagliptin in 5. The duration of treatment ranged from 12 weeks to 40 months. Forty-three trials were placebo-controlled and 28 used an active comparator, while nine trials included both placebo and active comparator arms. Active comparators included albiglutide, canagliflozin, empagliflozin, glipizide, glimepiride, metformin, voglibose, or thiazolidinediones. Of the 62 trials included in the meta-analysis, 61 were double blind trials.

### Risk ratio of fracture

**Figure 2:**
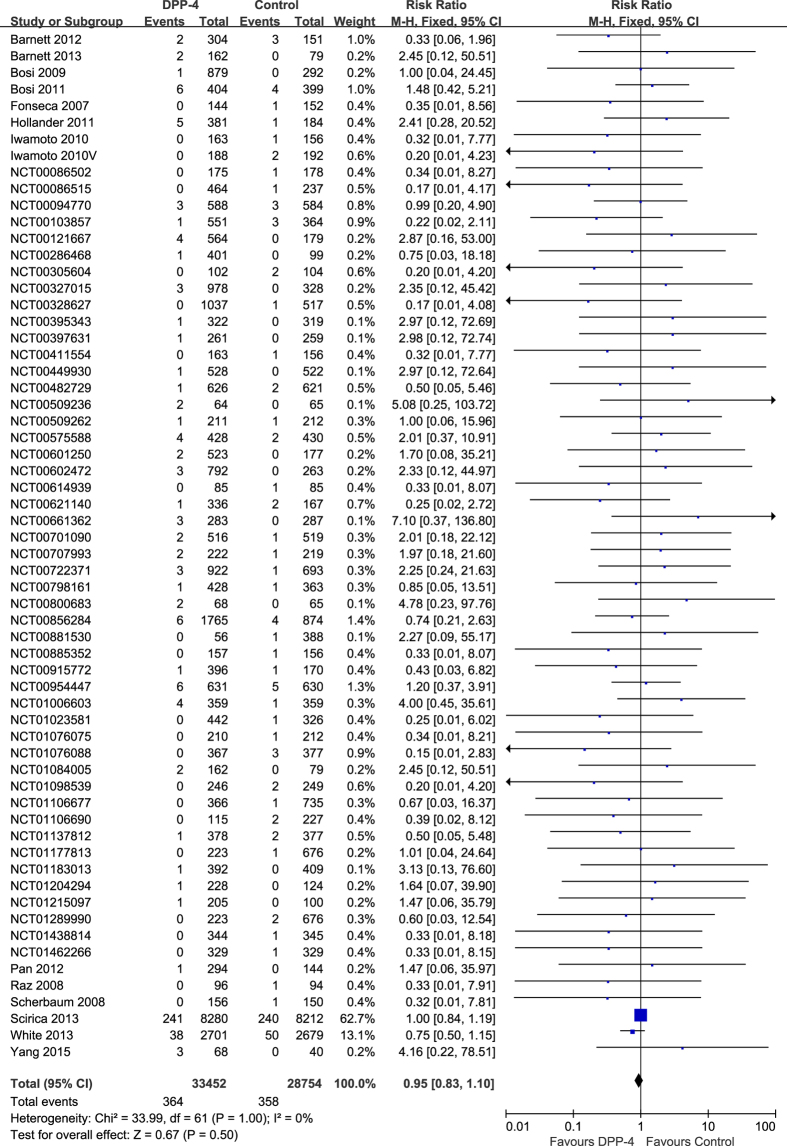
Risk of fractures between patients with type 2 diabetes treated with DPP-4 inhibitors or control.

A meta-analysis was performed to calculate the overall risk ratio (RR) of fracture associated with DPP-4 inhibitors versus control. Analysis of 62 trials showed that DPP-4 inhibitors were not associated with a significantly increased risk of fracture. The RR of fracture for patients treated with DPP-4 inhibitors compared with that for controls was 0.95 [95% confidence interval (CI) 0.83–1.10, P = 0.50), with insignificant heterogeneity (I^2^ = 0%) (Fig. 2). The evidence quality was moderate to high (Table S2).

### Subgroup analysis according to drug type

Subgroup analysis was performed to determine whether drug type had an effect on the RR of fracture with DPP-4 inhibitors. The RR of fracture with individual DPP-4 inhibitors was 0.79 (95% CI: 0.55–1.13, P = 0.19) for alogliptin (seven trials with 12,085 individuals, enrolling 53 patients with fracture in the experimental group and 61 patients with fracture in the control group), 1.25 (0.66–2.38, P = 0.50) for linagliptin (13 trials with 7638 individuals, enrolling 23 patients with fracture in the experimental group and 10 patients with fracture in the control group), 1.03 (0.87–1.22, P = 0.73) for saxagliptin (nine trials with 21,877 individuals, enrolling 266 patients with fracture in the experimental group and 248 patients with fracture in the control group), 0.66 (0.41–1.06, P = 0.08) for sitagliptin (27 trials with 17,907 individuals, enrolling 17 patients with fracture in the experimental group and 35 patients with fracture in the control group), 4.16 (0.22–78.51, P = 0.34) for anagliptin (one trial with 108 individuals, enrolling three patients with fracture in the experimental group and 0 patients with fracture in the control group) and 0.47 (0.13–1.78, P = 0.27) for vildagliptin (five trials with 2591 individuals, enrolling two patients with fracture in the experimental group and four patients with fracture in the control group). There were no statistically significant differences in the risk of fracture between individual DPP-4 inhibitors (P = 0.22) (Table 2). The evidence quality was moderate to high (Table S2).

### Subgroup analysis according to duration

Given the potential effect of duration of treatment on the association of DPP-4 inhibitors with risk of fracture, we performed a subgroup analysis stratified according to the length of follow-up. For a duration of ≥52 weeks with 41,641 participants, no statistically significant difference was observed between patients in the DPP4i and control groups (RR = 0.98, 95% CI, 0.84–1.13, P = 0.75), including 662 patients with fracture (332 in the experimental group and 330 in the control group). No significantly increased risk of fracture was observed for a duration of <52 weeks with 20,565 participants (RR = 0.78, 95% CI, 0.51–1.21, P = 0.28) including 60 patients with fracture (32 in the experimental group and 28 in the control group). There were no statistically significant differences in the risk of fracture according to the length of follow-up (P = 0.35) (Table 2). The evidence quality was moderate to high (Table S2).

### Subgroup analysis according to control regimen

Investigation of the effect of inhibitors according to the type of control (active treatment vs. placebo) did not suggest apparent differences (P = 0.76). In trials using active drug for comparison with 16,773 participants, the RR was 0.88 (95% CI: 0.56–1.39, P = 0.58), including 59 patients with fracture (24 in the experimental group and 35 in the control group). In trials using placebo for comparison with 47,953 participants, the RR was 0.95 (95% CI: 0.82–1.10, P = 0.48), including 674 patients with fracture (340 in the experimental group and 334 in the control group) (Table 2). The evidence quality was moderate to high (Table S2).

### Risk of specific fractures

Individual specific and non-specific fractures were listed in Table S3. There was no significant difference between the two groups in the incidence of specific fractures.

### Publication bias

No evidence of publication bias was detected for the RR of fracture in this study (Figure S1).

## Discussion

The effects of DPP-4 inhibitors on bone fractures in type 2 diabetes patients have not been well documented. Here, we performed an updated meta-analysis to provide a summary of current data. Analysis of 62 RCTs demonstrated that the use of DPP-4 inhibitors does not affect the risk of bone fracture compared with placebo or other antidiabetic medications in patients with type 2 diabetes. The results were consistent across subgroups defined by type of DPP-4 inhibitor, type of control, and length of follow-up.

Our results were in line with a recently published retrospective population-based cohort study that examined 216,816 patients and suggested that DPP-4 inhibitors were not associated with fracture risk compared with controls or other NIADs^9^. Our study was inconsistent with that of Monami *et al*.^8^, which showed a 40% reduction of fracture risk in DPP4-I users compared with patients taking other anti-diabetic drugs or placebo^24^,^25^,^26^. However, the positive effect observed in this study could be related to the limited number of trials included in the analysis. Compared with the study by Monami *et al*.^8^, our study has several strengths. First, we collected data from 62 randomized trials (N = 62,206), which together involved approximately three times as many patients as those included in the study by Monami et al. (N = 21,055)^8^. Second, we explored sources of heterogeneity with three priori subgroup hypotheses and the results remained robust.

Out results were largely influenced by a large RCT (N = 16,492) that compared saxagliptin with placebo and showed that the incidence of bone fracture was comparable between saxagliptin and placebo users^16^. However, the results remained robust after omitting that trial.

Glucagon-like peptide-1 (GLP-1) has been suggested to have a beneficial effect on bone^27^,^28^. The enzyme DPP-4 is involved in the degradation of GLP-1, and DPP-4 inhibitors are able to inhibit this process^9^. However, a recent meta-analysis highlighted that the use of GLP-1 receptor agonists does not modify the risk of bone fracture in patients with type 2 diabetes compared with the use of other antidiabetic medications^29^. Moreover, a recent *in vivo* study showed that MK-0626, a DPP-4 inhibitor, had neutral effects on cortical and trabecular bone in an animal model of type 2 diabetes, and MK-0626 did not alter osteoblast differentiation^30^. Thus, bone quality may be more important than bone density in predicting the increased risk for fractures in patients with type 2 diabetes^31^.

The present meta-analysis had several limitations. First, the duration of the trials included was not long enough to analyze the effects of DPP-4 inhibitors on the risk of bone fracture. We performed a subgroup analysis according to duration (≥52 weeks vs. <52 weeks) and found that the risk of fracture in different length of follow up were not significantly different. Second, fractures were not the primary endpoints in any of the included trials and were reported only as serious adverse events. Finally, no data could be obtained about gender and menopausal status. Therefore, trials with a longer follow-up duration and bone fracture as the primary endpoint are needed to further investigate the effects of DPP-4 inhibitors on fracture risk.

In summary, the current analysis suggested that the use of DPP-4 inhibitor does not decrease the risk of fracture in patients with type 2 diabetes. Given the negative effects of certain anti-diabetic drugs on bone, the results of the present study may be disappointing; however, a neutral effect on bone is still reassuring.

## Methods

### Data Sources and Searches

An extensive search of Medline, Embase, and Cochrane Central Register of Controlled Trials was performed by two of the investigators (J.F. and J.Z.). Data were collected on all randomized clinical trials in humans up to March 2016. Discrepancies in abstracted data between the reviewers were resolved by a third reviewer (Z.Z.). The search terms used were as follows: “DPP-4”, “dipeptidyl peptidase 4”, “alogliptin”, “linagliptin”, “saxagliptin”, “sitagliptin”, “vildagliptin”, “anagliptin”, and “dutogliptin”. The results of unpublished data were identified through a search of the www.clinicaltrials.gov website.

### Study Selection

The trials that met the following criteria were included in the analysis: (a) randomized clinical trials in type 2 diabetes patients; (b) duration of at least 12 weeks; (c) patients assigned to treatment with DPP-4 inhibitors compared with placebo or active drugs; (d) data on bone fracture was available; and (f) trials with two zero events were excluded from the analysis.

### Data Extraction and Quality Assessment

The following information was extracted independently from eligible RCTs by two of the investigators (Y.H. and C.G.): author’s name, year of publication, study design, sample size, number of treatment groups, length of follow-up, mean age, and registry number. In addition, for trials in which fracture data had not been published previously, the investigators abstracted the relevant numbers from their previously established databases of adverse events. The quality of included trials was assessed using the Jadad score^32^, which was only used for descriptive purposes. Any discrepancies in abstracted data between the reviewers were resolved by a third reviewer (Z.Z.).

### Data analysis

The meta-analysis was performed following the PRISMA checklist^33^. The main outcome was bone fracture reported as a serious adverse event. Trials were pooled using the Mantel-Haenszel method to calculate RRs and their 95% CIs. P < 0.05 was considered significant. For studies reporting zero fracture events in a treatment or control arm, a classic half-integer continuity correction was used to calculate the RR and variance. Heterogeneity between studies was assessed by using the χ2 test and the I^2^ statistic. Selection of the fixed- or random-effects model depended on the result of the Cochrane’s *Q* test. An I^2^ value of 50% was considered to indicate significant heterogeneity between trials^34^. A fixed effects model was applied if there was no statistical heterogeneity among the studies; otherwise, the random effects model was used^34^. Pre-defined subgroup analyses were performed for trials that included different types of DPP-4 inhibitors (alogliptin, linagliptin, saxagliptin, sitagliptin, anagliptin, and vildagliptin), different types of control (active treatment vs. placebo), and different lengths of follow-up (≥52 weeks vs. <52 weeks). Finally, publication bias was evaluated through funnel plots. Meta-analyses were performed using Review Manager 5.1 software. The criteria of the Grading of Recommendations Assessment, Development and Evaluation were used to evaluate the quality of evidence by outcome.^35^

## Additional Information

**How to cite this article**: Fu, J. *et al.* Dipeptidyl peptidase-4 inhibitors and fracture risk: an updated meta-analysis of randomized clinical trials. *Sci. Rep.* **6**, 29104; doi: 10.1038/srep29104 (2016).

## Supplementary Material

Supplementary Information

## Figures and Tables

**Table 1:** Characteristics of studies included in primary analysis.

Study	NCT code	DPP-4	Comparator(s)	N. of patients		Duration (weeks)	Age (years)	HbA1c (%)	Fracture	
				DPP-4	Control				DPP-4	Control
Bosi^10^	NCT00432276	ALOG	Pioglitazone	404	399	52	55	8.3	6	4
NCT00286468	NCT00286468	ALOG	Placebo	401	99	26	57	8	1	0
NCT01023581	NCT01023581	ALOG	Placebo/metformin	450	334	26	53.5	8.5	0	1
NCT00856284	NCT00856284	ALOG	Glipizide	1765	874	104	55.4	7.6	6	4
NCT00328627	NCT00328627	ALOG	Placebo/pioglitazone	1037	517	26	54.4	8.6	0	1
NCT00707993	NCT00707993	ALOG	Glipizide	222	219	52	69.9	NR	2	1
White^11^	NCT00968708	ALOG	Placebo	2701	2679	40 months	60.9	8	38	50
NCT01183013	NCT01183013	LINA	Placebo	392	409	54	57.1	8.11	1	0
NCT00915772	NCT00915772	LINA	Placebo/metformin	171	170	54	55.8	7.5	1	1
NCT00798161	NCT00798161	LINA	Placebo/metformin	428	363	24	55.2	8.91	1	1
NCT01438814	NCT01438814	LINA	Placebo	344	345	14	53	NR	0	1
NCT00601250	NCT00601250	LINA	Placebo	523	177	24	56.5	8.08	2	0
NCT01084005	NCT01084005	LINA	Placebo	162	79	24	74.9	7.78	2	0
NCT00954447	NCT00954447	LINA	Placebo	631	630	52	60	8.3	6	5
NCT00602472	NCT00602472	LINA	Placebo	792	263	24	58.1	8.14	3	0
NCT00800683	NCT00800683	LINA	Placebo	68	65	52	64.4	8.2	2	0
NCT00621140	NCT00621140	LINA	Placebo	336	167	24	55.7	8	1	2
NCT01204294	NCT01204294	LINA	Metformin	228	124	52	60.9	NR	1	0
NCT01215097	NCT01215097	LINA	Placebo	205	100	24	55.5	7.99	1	0
Barnett^12^	NCT01084005	LINA	Placebo	162	79	24	74.9	7.78	2	0
Barnett^13^	NCT00757588	SAXA	Placebo	304	151	52	57.2	8.7	2	3
Hollander^14^	NCT00295633	SAXA	Placebo	381	184	24	54	8	5	1
Scirica^15^	NCT01107886	SAXA	Placebo	8280	8212	2.9 years	65	NR	241	240
NCT01006603	NCT01006603	SAXA	Glimepiride	359	359	52	72.6	NR	4	1
NCT00121667	NCT00121667	SAXA	Placebo	564	179	206	54.57	8.1	4	0
NCT00575588	NCT00575588	SAXA	Glipizide	428	430	52	57.55	7.7	4	2
NCT00614939	NCT00614939	SAXA	Placebo	85	85	52	66.5	NR	0	1
NCT00327015	NCT00327015	SAXA	Placebo/metformin	643	328	76	52	9.5	3	0
NCT00661362	NCT00661362	SAXA	Placebo	283	287	24	54	7.9	3	0
NCT00509236	NCT00509236	SITA	Glipizide	64	65	54	59.5	NR	2	0
NCT01076088	NCT01076088	SITA	Placebo/metformin	367	377	24	52.7	8.7	0	3
NCT00509262	NCT00509262	SITA	Glipizide	211	212	54	64.2	7.8	1	1
NCT01076075	NCT01076075	SITA	Pioglitazone	210	212	54	54.9	8.4	0	1
NCT00885352	NCT00885352	SITA	Placebo	157	156	26	56.1	8.7	0	1
NCT00395343	NCT00395343	SITA	Placebo	322	319	24	57.8	8.7	1	0
NCT00722371	NCT00722371	SITA	Placebo/pioglitazone	691	693	54	57	NR	3	1
NCT01462266	NCT01462266	SITA	Placebo	329	329	24	58.8	NR	0	1
NCT00305604	NCT00305604	SITA	Placebo	102	104	24	71.9	7.8	0	2
NCT00411554	NCT00411554	SITA	Voglibose	163	156	12	60.7	7.8	0	1
NCT00103857	NCT00103857	SITA	Placebo/ metformin	372	540	104	53.4	9	1	3
NCT01177813	NCT01177813	SITA	Empagliflozin	223	448	31	55	NR	0	1
NCT00449930	NCT00449930	SITA	Metformin	528	522	24	56	7.3	1	0
NCT00701090	NCT00701090	SITA	Glimepiride	516	519	30	56.3	7.5	2	1
NCT00086515	NCT00086515	SITA	Glipizide	464	237	24	54.5	8	0	1
NCT01098539	NCT01098539	SITA	Albiglutide	246	249	26	63.3	NR	0	2
NCT00086502	NCT00086502	SITA	Placebo	175	178	24	56.2	8	0	1
NCT00094770	NCT00094770	SITA	Glipizide	588	584	104	56.7	7.7	3	3
NCT01289990	NCT01289990	SITA	Placebo/empagliflozin	223	223	76	55.6	NR	0	2
NCT00482729	NCT00482729	SITA	Placebo	625	621	44	49.7	9.87	1	2
NCT00397631	NCT00397631	SITA	Placebo	261	259	24	50.9	9.5	1	0
NCT01106677	NCT01106677	SITA	Canagliflozin	366	735	52	55.4	NR	0	1
NCT01137812	NCT01137812	SITA	Canagliflozin	378	377	52	56.5	NR	1	2
NCT01106690	NCT01106690	SITA	Canagliflozin	115	227	52	57.4	NR	0	2
NCT00881530	NCT00881530	SITA	Placebo	56	56	78	58.6	NR	0	1
Iwamoto^16^	NR	SITA	Voglibose	163	156	12	60.7	7.8	0	1
Raz^17^	NCT00337610	SITA	Placebo	96	94	30	54.8	9.2	0	1
Bosi^18^	NCT00468039 NCT00382096	VILDA	Placebo	292	292	24	52.8	8.65	1	0
Fonseca^19^	NCT00099931	VILDA	Placebo	144	152	24	59.2	8.4	0	1
Iwamoto^20^	NR	VILDA	Voglibose	188	192	12	60.3	7.5	0	2
Pan^21^	NR	VILDA	Placebo	294	144	24	54.2	8.05	1	0
Scherbaum^22^	NCT00101712	VILDA	Placebo	156	155	52	63.3	6.7	0	1
Yang^23^	NR	ANAG	Placebo	60	48	24	56.2	7.14	3	0

ALOG, alogliptin; LINA, linagliptin; SAXA, saxagliptin; SITA, sitagliptin; VILDA, vildagliptin; ANAG, anagliptin;NR, nor reported.

**Table 2:** Risk ratio of fracture by subgroup analyses.

Subgroup	Studies n	No. of fracture		No. of participants		Risk ratio (95% CI)	P Value	
		DPP-4	Control	DPP-4	Control		RR	Group difference
Overall Individual DPP-4	62	364	358	33452	28754	0.95 (0.82, 1.10)	0.50	NA
Alogliptin	7	53	61	6972	5113	0.79 (0.55, 1.14)	0.20	0.37
Linagliptin	13	23	10	4667	2971	1.19 (0.60, 2.38)	0.62	
Saxagliptin	9	266	248	11662	10215	1.02 (0.86, 1.21)	0.84	
Sitagliptin	27	17	35	8422	9485	0.67 (0.39, 1.15)	0.15	
Anagliptin	1	3	0	68	40	4.16 (0.22, 78.51)	0.34	
Vildagliptin	5	2	4	1661	930	0.50 (0.12, 2.05)	0.33	
Duration								
≥52 weeks	28	332	330	21645	19996	0.97 (0.83, 1.13)	0.69	0.37
<52 weeks	34	32	28	11807	8758	0.76 (0.46, 1.27)	0.29	
Comparators								
Active drug	28	24	35	7594	9179	0.91 (0.54, 1.52)	0.71	0.88
Placebo	43	340	334	26235	21718	0.95 (0.81, 1.10)	0.44	

NA, not applicable.
