# Supplementary material for: Dipeptidyl peptidase-4 inhibitors and fracture risk: an updated meta-analysis of randomized clinical trials
Source: Sci Rep. 2016 Jul 7;6:29104. doi: 10.1038/srep29104 (PMC4935882; doi:10.1038/srep29104)
Supplement: Supplementary Information [file srep29104-s1.pdf]

Dipeptidyl peptidase-4 inhibitors and fracture risk: an updated meta-analysis of randomized clinical trials

Jianying Fu[1*], Jianhong Zhu[2*], Yehua Hao[1], Chongchong Guo[1] and Zhikun Zhou[1]

Jianying, Fu, Guangdong Medical University, 523808, 404978306@qq.com.

Jianhong, Zhu, Sun Yat-Sen Memorial Hospital, Guangzhou, 510120, 645582117@qq.com.

Yehua, Hao, Guangdong Medical University, 523808, 1528735424@qq.com.

Chongchong, Guo, Guangdong Medical University, 523808, 942826128@qq.com.

Zhikun, Zhou, Guangdong Medical University, 523808, zhouzhkun@126.com, 18928033088, 0756-2656022.

Supplementary figure 1. Funnel plot for publication bias

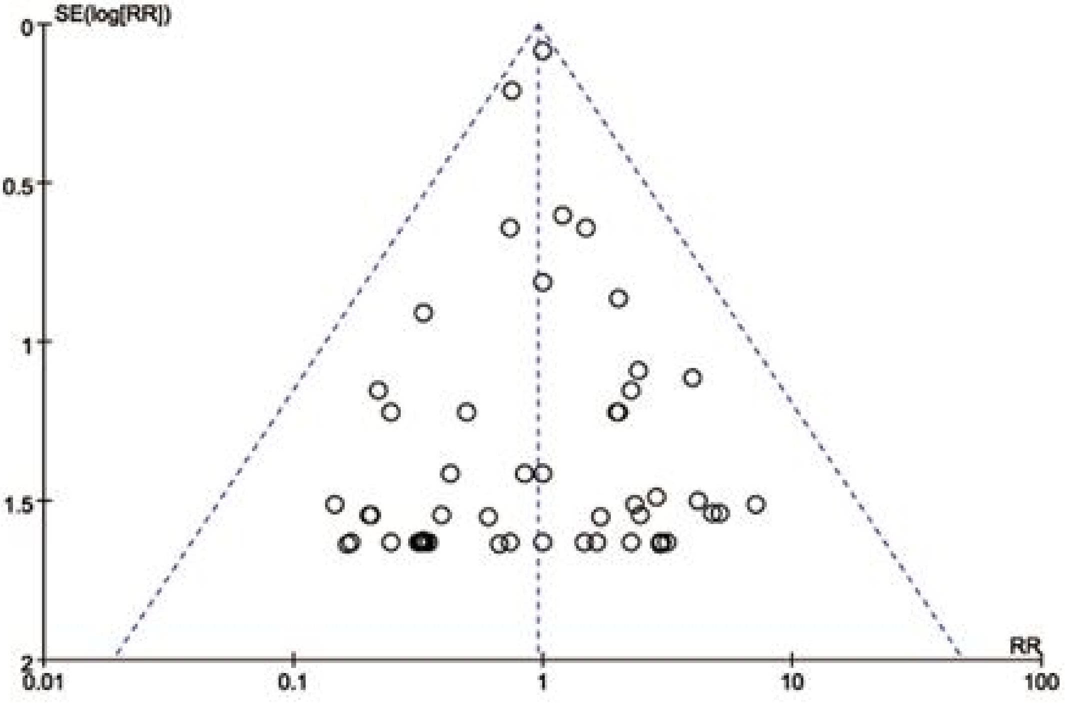

Supplemental table 1.Quality assessment of the included studies

Study	NCT code	Description of Randomization	Description of Allocation	Description of Blinding	Reporting of Drop out	Intention to treat
Alogliptin						
Bosi 2011	NCT00432276	NA	Y	Y	Y	Y
NCT00286468	NCT00286468	NA	NA	Y	Y	Y
NCT01023581	NCT01023581	NA	NA	Y	Y	Y
NCT00856284	NCT00856284	NA	NA	Y	Y	Y
NCT00328627	NCT00328627	NA	NA	Y	Y	Y
NCT00707993	NCT00707993	NA	NA	Y	Y	Y
White 2013	NCT00968708	Y	Y	Y	Y	Y
NCT01183013	NCT01183013	NA	NA	Y	Y	Y
NCT00915772	NCT00915772	NA	NA	Y	Y	Y
NCT00798161	NCT00798161	NA	NA	Y	Y	Y
NCT01438814	NCT01438814	NA	NA	Y	Y	Y
NCT00601250	NCT00601250	NA	NA	Y	Y	Y
NCT01084005	NCT01084005	NA	NA	Y	Y	Y
NCT00954447	NCT00954447	NA	NA	Y	Y	Y
NCT00602472	NCT00602472	NA	NA	Y	Y	Y
NCT00800683	NCT00800683	NA	NA	Y	Y	Y
NCT00621140	NCT00621140	NA	NA	Y	Y	Y
NCT01204294	NCT01204294	NA	NA	N	N	Y
NCT01215097	NCT01215097	NA	NA	Y	Y	Y
Barnett 2013	NCT01084005	Y	Y	Y	Y	Y
Saxagliptin						
Barnett 2012	NCT00757588	Y	Y	Y	Y	Y
Hollander 2011	NCT00295633	NA	Y	Y	Y	Y
Scirica 2013	NCT01107886	Y	Y	Y	Y	Y
NCT01006603	NCT01006603	NA	NA	Y	Y	Y
NCT00121667	NCT00121667	NA	NA	Y	Y	Y
NCT00575588	NCT00575588	NA	NA	Y	Y	Y
NCT00614939	NCT00614939	NA	NA	Y	Y	Y
NCT00327015	NCT00327015	NA	NA	Y	Y	Y
NCT00661362	NCT00661362	NA	NA	Y	Y	Y
Sitagliptin						
NCT00509236	NCT00509236	NA	NA	Y	Y	Y
NCT01076088	NCT01076088	NA	NA	Y	Y	Y
NCT00509262	NCT00509262	NA	NA	Y	Y	Y
NCT01076075	NCT01076075	NA	NA	Y	Y	Y
NCT00885352	NCT00885352	NA	NA	Y	Y	Y
NCT00395343	NCT00395343	NA	NA	Y	Y	Y
NCT00722371	NCT00722371	NA	NA	Y	Y	Y

NCT01462266	NCT01462266	NA	NA	Y	Y	Y
NCT00305604	NCT00305604	NA	NA	Y	Y	Y
NCT00411554	NCT00411554	NA	NA	Y	Y	Y
NCT00103857	NCT00103857	NA	NA	Y	Y	Y
NCT01177813	NCT01177813	NA	NA	Y	Y	Y
NCT00449930	NCT00449930	NA	NA	Y	Y	Y
NCT00701090	NCT00701090	NA	NA	Y	Y	Y
NCT00086515	NCT00086515	NA	NA	Y	Y	Y
NCT01098539	NCT01098539	NA	NA	Y	Y	Y
NCT00086502	NCT00086502	NA	NA	Y	Y	Y
NCT00094770	NCT00094770	NA	NA	Y	Y	Y
NCT01289990	NCT01289990	NA	NA	Y	Y	Y
NCT00482729	NCT00482729	NA	NA	Y	Y	Y
NCT00397631	NCT00397631	NA	NA	Y	Y	Y
NCT01106677	NCT01106677	NA	NA	Y	Y	Y
NCT01137812	NCT01137812	NA	NA	Y	Y	Y
NCT01106690	NCT01106690	NA	NA	Y	Y	Y
NCT00881530	NCT00881530	NA	NA	Y	Y	Y
Iwamoto 2010		Y	Y	Y	Y	Y
Raz 2008	NCT00337610	Y	Y	Y	Y	Y
Vildagliptin						
Bosi 2009	NCT00468039 NCT00382096	NA	Y	Y	Y	Y
Fonseca 2007	NCT00099931	NA	Y	Y	Y	Y
Iwamoto 2010		NA	Y	Y	Y	Y
Pan 2012		NA	Y	Y	Y	Y
Scherbaum 2008	NCT00101712	NA	Y	Y	Y	Y
Yang 2015		NA	Y	Y	Y	Y

NA, Not Adequately described; Y, yes; N, no

Supplementary table 2. the Grading of Recommendations Assessment, Development and Evaluation.

GRADE evidence profile: evaluating DPP-4 inhibitors versus placebo or other anti-diabetic medications in patients with type 2 diabetes								
Participants (studies)	Risk of bias	Inconsistency	Indirectness	Imprecision	Publication bias	Overall quality of evidence	Relative effect (95% CI)	Anticipated absolute effects
All fracture								
62206 (62 studies)	Serious	No serious	No serious	No serious	Undetected	Moderate	RR 0.95 (0.83 to 1.1)	1 fewer per 1000 (from 2 fewer to 1 more)
Drug								
62206 (62 studies)	Serious	No serious	No serious	No serious	Undetected	Moderate	RR 0.95 (0.82 to 1.1)	1 fewer per 1000 (from 2 fewer to 1 more)
Drug - Alogliptin								
12085 (13 studies)	Serious	No serious	No serious	No serious	Undetected	Moderate	RR 0.79 (0.55 to 1.14)	3 fewer per 1000 (from 5 fewer to 2 more)
Drug - Linagliptin								
7638 (13 studies)	Serious	No serious	No serious	No serious	Undetected	Moderate	RR 1.19 (0.6 to 2.38)	1 more per 1000 (from 1 fewer to 5 more)
Drug - saxagliptin								
21877 (9 studies)	Serious	No serious	No serious	No serious	Undetected	Moderate	RR 1.02 (0.86 to 1.21)	0 more per 1000 (from 3 fewer to 5 more)
Drug - Sitagliptin								
17907 (27 studies)	Serious	No serious	No serious	No serious	Undetected	Moderate	RR 0.67 (0.39 to 1.15)	1 fewer per 1000 (from 2 fewer to 1 more)
Drug - anagliptin								
108 (1 study)	Serious	No serious	No serious	No serious	Undetected	Moderate	RR 4.16 (0.22 to 78.51)	0 per 1000
Drug - Vildagliptin								
2591 (5 studies)	Serious	No serious	No serious	No serious	Undetected	Moderate	RR 0.5 (0.12 to 2.05)	2 fewer per 1000 (from 4 fewer to 5 more)
Control								
64726 (62 studies)	Serious	No serious	No serious	No serious	Undetected	Moderate	RR 0.94 (0.82 to 1.09)	1 fewer per 1000 (from 2 fewer to 1 more)
Control - ACTIVE								
16773 (28 studies)	Serious	No serious	No serious	No serious	Undetected	Moderate	RR 0.91 (0.54 to 1.52)	0 fewer per 1000 (from 2 fewer to 2 more)
Control - PBO								

47953<br>(43 studies)	Serious	No serious	No serious	No serious	Undetected	Moderate	RR 0.95<br>(0.81 to 1.1)	1 fewer per 1000<br>(from 3 fewer to 2 more)
DURATION								
62206<br>(62 studies)	Serious	No serious	No serious	No serious	Undetected	Moderate	RR 0.95<br>(0.82 to 1.1)	1 fewer per 1000<br>(from 2 fewer to 1 more)
DURATION - ≥52 weeks								
41641<br>(28 studies)	Serious	No serious	No serious	No serious	Undetected	Moderate	RR 0.97<br>(0.83 to 1.13)	0 fewer per 1000<br>(from 3 fewer to 2 more)
DURATION - ＜ 52 weeks								
20565<br>(34 studies)	Serious	No serious	No serious	No serious	Undetected	Moderate	RR 0.76<br>(0.46 to 1.27)	1 fewer per 1000<br>(from 2 fewer to 1 more)

Supplemental table 3. Fracture events by locations

Location of fracture	Events of fracture	
	DPP4	Control
Lower limb fracture	122	108
Foot	18	16
Femur	21	21
Ankle	15	21
Tibia	8	9
Femoral neck	14	8
Fibula	6	6
Humerus	13	9
Hip	12	12
Patella	9	4
Ischium	2	0
Pelvic	3	1
Acetabulum	1	1
Upper limb fracture	43	41
Hand	8	10
Radius	8	12
Wrist	11	6
Ulna	4	1
Forearm	1	2
Scapula	1	1
Clavicle	2	1
Unspecified upper limb	8	8
Spine fracture	16	23
Spinal compression	6	8
Pubis	3	5
Lumbar vertebral	5	6
Cervical vertebral	0	3
Chest fracture	16	17
Rib	11	13
Sternal	1	3
Thoracic	4	1
Face	4	4
Facial bones	4	4
Unspecified fracture	160	165
